# Supplementary figures and images for: Transcriptional profiling of peripheral blood mononuclear cells identifies inflammatory phenotypes in Ataxia Telangiectasia
Source: Orphanet J Rare Dis. 2024 Feb 14;19:67. doi: 10.1186/s13023-024-03073-5 (PMC10870445; doi:10.1186/s13023-024-03073-5)

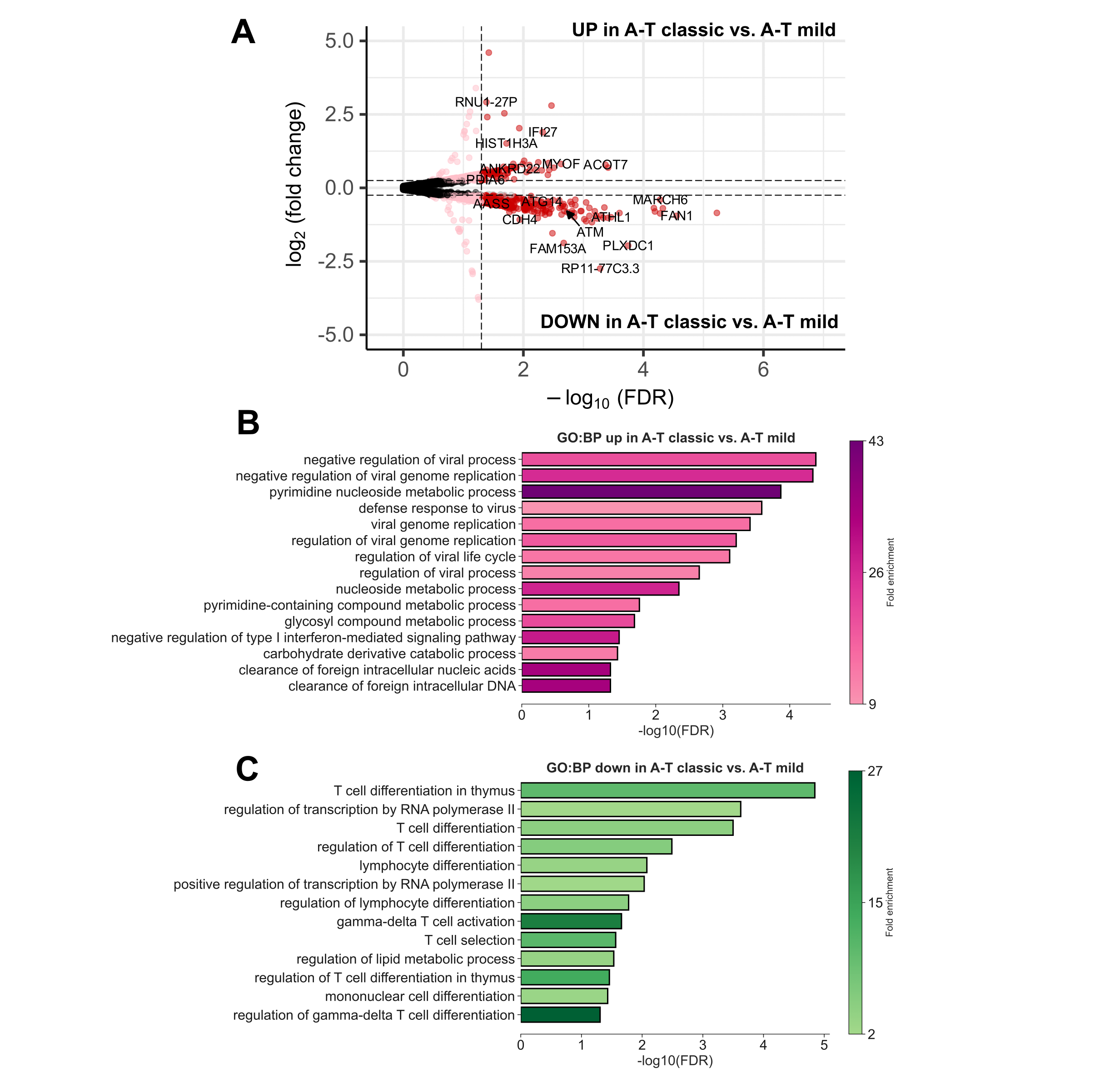

Supplement: Supplementary file 1 — Supplementary Material 1 [file 13023_2024_3073_MOESM1_ESM.png]

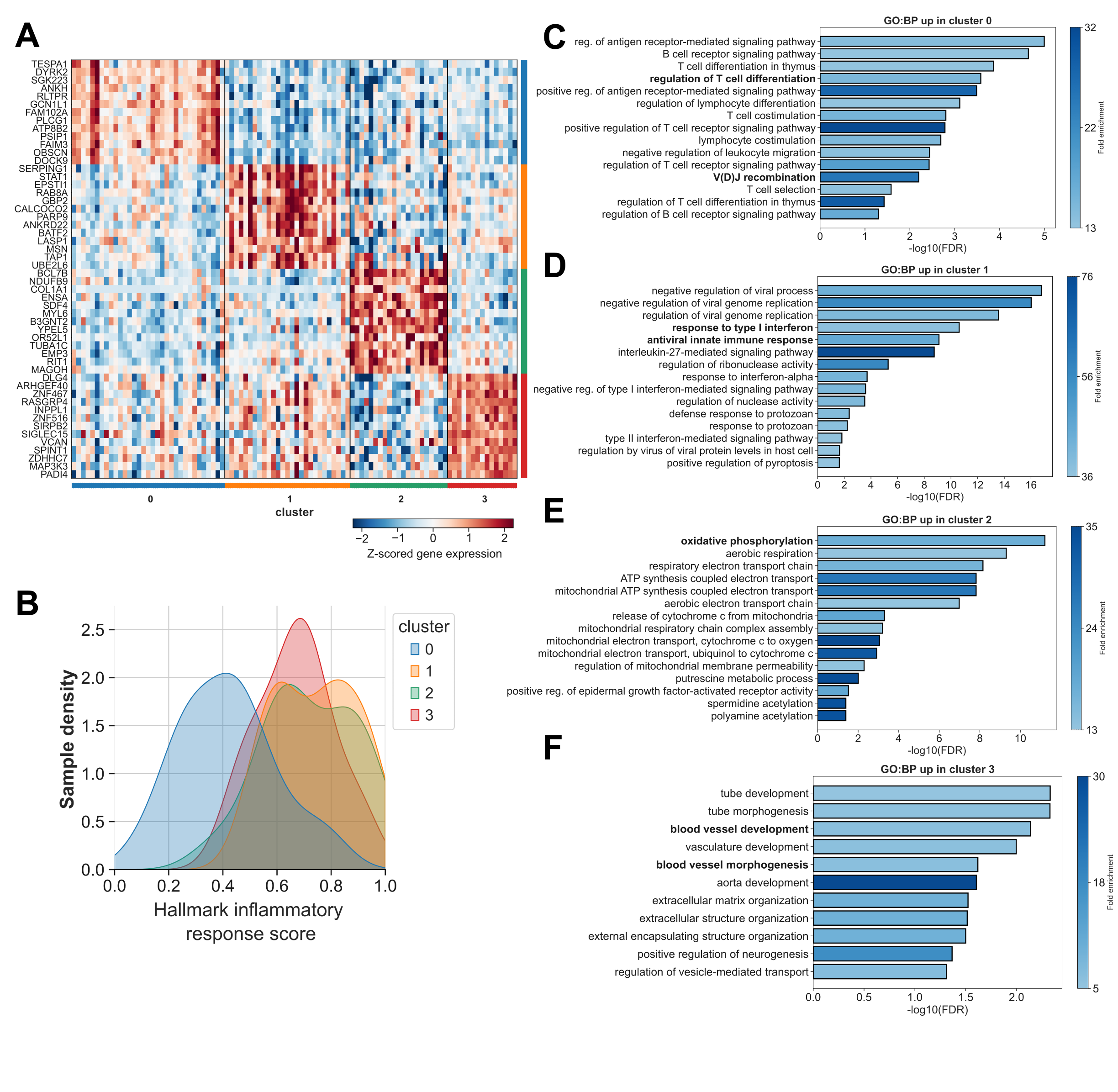

Supplement: Supplementary file 2 — Supplementary Material 2 [file 13023_2024_3073_MOESM2_ESM.png]
